# Variations in Perceptions of Well-Being within Families of Youths with Intellectual Disabilities in Saudi Arabia

**DOI:** 10.3390/children11060644

**Published:** 2024-05-27

**Authors:** Ghaleb H. Alnahdi

**Affiliations:** Special Education Department, College of Education, Prince Sattam bin Abdulaziz University, Alkharj 11902, Saudi Arabia; g.alnahdi@psau.edu.sa

**Keywords:** intellectual disability, family quality of life, cultural influences in health, Saudi Arabia, parental perceptions, disabilities studies

## Abstract

This study investigates the family well-being among Saudi Arabian families with youth who have an intellectual disability. A sample of 148 family members, including parents and other relatives, was surveyed on emotional well-being, family interactions, and parenting. This study aimed to explore the unique challenges and dynamics within these families, providing insights into how an intellectual disability in a youth affects the family unit. The research highlights a gap in understanding the specific impacts of intellectual disability on family life in the Saudi context. Key findings include variations in family quality of life perceptions among different family members, with fathers showing distinct levels of satisfaction. This study contributes to the development of culturally sensitive support strategies and policies, emphasizing the need for targeted interventions to enhance the well-being of these families in Saudi Arabia.

## 1. Introduction

Individuals with disabilities face distinct challenges and demands. These include objective outcomes such as unemployment, as highlighted by Ohl et al. [1], and a lower quality of life (QOL). The World Health Organization’s definition of QOL encompasses self-perception in life in relation to goals, expectations, standards, and concerns [2]. Studies, such as the meta-analysis by van Heijst and Geurts [3], reveal a considerable QOL gap between individuals with and without disabilities.

The impact of a disability extends beyond the individual to encompass the entire family unit [4]. Various challenges arise, such as health management, caregiving responsibilities, and the economic burdens associated with supporting a family member with a disability [5,6,7]. Recent studies have expanded to include the family’s perspective in assessing the QOL of individuals with disabilities [8], reflecting a broader approach to incorporating their needs into service provision. Families dealing with disabilities typically could face heightened stress, depression, or reduced satisfaction with life, which might be influenced by the severity of the disability [9,10,11,12]. While previous research has broadly examined some of the challenges faced by families of individuals with intellectual disabilities, the cultural uniqueness of these experiences in Saudi Arabia remains underexplored. This study aims to fill this gap by examining the impact of intellectual disabilities within the unique socio-cultural context of Saudi families. Specifically, this study delves into how traditional roles, cultural expectations, and social norms in Saudi Arabia shape family dynamics and caregiving practices, which are pivotal to developing targeted and culturally sensitive interventions.

Our research focuses on the profound impact that intellectual disabilities within a family have on the well-being and emotional health of all its members. We investigate how these disabilities affect family dynamics, including caregiving challenges, financial burdens, and emotional stress. By recognizing and addressing these issues through supportive interventions, we aim to enhance the overall quality of life for these families, highlighting the importance of understanding their unique needs [13,14]. It is essential to approach the quality of life of families with developmental disabilities from their perspective, serving as a crucial preliminary step toward providing effective support.

This study aims to understand the impact of having a member with an intellectual disability on the quality of life (QOL) of families in Saudi Arabia. Although there have been global research efforts to understand the effects of such disabilities on families, including their overall QOL and family dynamics, a significant gap remains in the context of Saudi Arabia. Our research seeks to bridge this gap by providing insights into the unique challenges and experiences faced by these families. This understanding is crucial for the development of culturally appropriate support strategies and policies, aimed at enhancing the well-being of individuals with intellectual disabilities and their families in Saudi Arabia. Our selection of disability severity, child age, and relation to the child as independent variables in this study draws from foundational research [15] that established that disability severity significantly predicts parental satisfaction with family quality of life, underscoring the need to explore how different levels of disability impact family dynamics. Bhopti et al. [16] further highlighted the evolving nature of caregiving challenges as the child ages, pointing to increased hardships but also the potential for positive adaptations that support family quality of life. These insights guide our focus on these variables to delve into how they influence the quality of life within Saudi families, aiming to uncover specific challenges and opportunities for support in this cultural context. Additionally, the inclusion of the child’s relation as an independent variable aims to help us understand the differential impact of caregiving responsibilities and satisfaction levels among various family members, recognizing that each member’s experience and contribution to caregiving may vary significantly.

In understanding our study’s relevance, it is crucial to consider both the unique circumstances of Saudi Arabia and the wider body of research concerning families with a member with a disability. According to the General Authority of Statistics in Saudi Arabia [17], approximately 7.1% of the population is estimated to have a disability in general. This prevalence has diverse effects on the quality of life of families, with significant challenges being highlighted in different studies [18,19,20]. Collectively, these studies underscore a diminished quality of life among families of children with disabilities, particularly affecting social, environmental, and leisure activities. This phenomenon is paralleled in findings from other Arab countries, such as Egypt, where studies by Ahmad [21] and Darwish [22] reveal comparable strains on caregivers’ QoL, indicating broader regional trends. These findings affirm that the pressures faced by families of children with disabilities in Saudi Arabia align with those in other regional contexts, underlining the universal challenges of caregiving across different settings, yet also pointing towards unique aspects within the Saudi context that necessitate culturally sensitive interventions. In the context of Saudi Arabia, several unique socio-cultural and economic aspects could influence the experiences of families with members who have intellectual disabilities. These include traditional family roles where fathers often carry financial and decision-making burdens, deeply rooted social norms that prioritize family privacy, and specific challenges associated with accessing disability services due to regional disparities in service provision. Moreover, the societal stigma associated with disability can significantly impact family dynamics, influencing everything from marital prospects to social isolation. Understanding these unique cultural and social dynamics is crucial for developing effective support systems tailored to the needs of Saudi families.

This study fills a notable gap in the literature by exploring the family quality of life (FQOL) within the unique cultural, social, and economic context of Saudi Arabia. Previous research has predominantly focused on Western contexts, leaving a critical understanding gap in the Middle East. By examining the experiences of Saudi families, our study provides insights into the specific challenges and opportunities faced by these families, contributing to the development of culturally sensitive support strategies and policies. This study is exploratory, aiming to investigate the unique challenges and dynamics within Saudi Arabian families of youths with intellectual disabilities. It seeks to uncover how these challenges impact the overall family quality of life and to provide insights that could guide the development of culturally sensitive support strategies. The primary research question explores whether there is an association between the severity of a child’s disability, the child’s age, and the relationship to the child with a disability, and how these factors influence the family’s perception of quality of life in the presence of a youth with an intellectual disability in Saudi Arabia. This investigation aims to uncover the unique challenges faced by these families and identify the necessary support systems to improve their well-being

## 2. Methodology

### 2.1. Instrument 

The study conducted was a cross-sectional survey designed to evaluate the family quality of life among Saudi Arabian families with youths who have an intellectual disability. This design was chosen to assess the current state and perceptions at a specific point in time without manipulating any variables. In our study, we employed the Beach Center Family Quality of Life Scale to assess the quality of life among families with a youth who has an intellectual disability [23,24,25,26]. The Beach Center’s Family Quality of Life Scale (BC-FQOL) is notably the most commonly used scale in studies examining family quality of life (FQOL), particularly in the context of families with members that have intellectual disabilities. This scale has been employed in 49% of 120 relevant studies in a recent compressive review study [27], highlighting its widespread acceptance and utilization in diverse cultural contexts across 27 countries (for example: [15,28,29]). The BC-FQOL comprises 25 items spanning five critical FQOL subdomains: family interaction, parenting, emotional well-being, physical/material well-being, and disability-related support. Its wide applicability is further evidenced by its translation and adaptation for use in various countries, where it has been rigorously tested for its psychometric properties.

In this study, we focused on three specific domains of the scale: emotional well-being, family interactions, and parenting. These domains were chosen for their relevance in capturing the key aspects of family life that are most impacted by the presence of a youth with a disability. The emotional well-being domain evaluates the overall emotional and psychological health of the family members. The family interactions domain assesses the quality and nature of relationships and interactions within the family. Lastly, the parenting domain focuses on the experiences and challenges related to raising a child with a disability. By concentrating on these three domains, our study aimed to gain a deeper understanding of the nuanced ways in which a youth’s intellectual disability affects the family’s quality of life. It is important to elucidate that our selection was informed by the foundational work of Alnahdi [30], which emphasized the multidimensionality of the Beach Center Family Quality of Life (BCFQOL) scale. Alnahdi’s study provided statistical evidence, via Rasch analysis, underscoring the unidimensional nature of each of the subscales, thereby validating the independent use of each subscale to assess distinct dimensions of family quality of life. Alnahdi’s findings support the notion that each domain within the BCFQOL can stand alone as a unidimensional scale, enabling focused analysis and interpretation of data within these specific areas of family life. This approach not only aligns with the methodological rigor established by previous research but also enhances our study’s capacity to address nuanced aspects of family dynamics, thereby contributing valuable insights into the multidimensional nature of family quality of life. In addition, the construct and convergent validity were supported by the findings of Alnahdi et al. [23], the reliability of the overall scale was (0.965), and the subscales ranged from 0.854 to 0.946. 

### 2.2. Sample 

The sample of this study was 148 relatives of youth with intellectual disabilities. Table 1 shows the descriptive statistics of the participants in this study, divided into two categories: the relationship of the family member to the youth with a disability, and the severity of the youth’s disability. For family relationships, mothers accounted for 29.7% of the total, fathers represented 41.2%, and other relatives (like brothers, sisters, and grandparents) comprised 29.1% of the total. In terms of the severity of the disability in the youth, 29.7% had a mild disability, 54.7% had a moderate disability, and 15.5% had a severe disability. Around 71% of parents in these families are still together with the youth with ID. The sample included individuals with disabilities, aged between 12 and 30 years. This broad age range encompasses both the adolescent and young adult demographics, offering valuable insights into the family’s quality of life during these critical developmental stages. The sampling method employed in this study was purposive sampling. This approach was chosen to specifically target families of youths with intellectual disabilities. Research assistants collaborated with local educational institutions and community centers to identify potential participants who met the study criteria. This method ensured that the sample was representative of the population of interest, focusing on those directly impacted by the issues under investigation.

Our study’s sample was recruited with the assistance of research assistants, who played a key role in contacting families of youths with disabilities to participate. The research assistants worked closely with the school administrations to contact families of youths with disabilities. An electronic version of the study materials was distributed through these schools, allowing for efficient distribution and collection of data. This method facilitated a streamlined process of gathering responses from the families, ensuring comprehensive coverage and high participation rates. The entire process, from the initial contact to the final collection of data, was completed within about nine weeks in March and April 2021, ensuring a rapid yet thorough approach to assembling a representative sample for our study.

This study included family members (parents, siblings, and other relatives) of youths aged between 12 and 30 years with a diagnosed intellectual disability. Inclusion criteria required participants to be directly involved in the caregiving process. Exclusion criteria included families without a formal diagnosis of intellectual disability and those residing outside the specified geographic area to maintain focus on the targeted community and cultural context.

This study adhered to strict ethical standards under the ethical guidelines and approval from Prince Sattam bin Abdulaziz University’s IRB. Informed consent was obtained from all participants, who were briefed on their rights to confidentiality, anonymity, and voluntary participation. 

## 3. Results

The results of this study intricately align with its aim to investigate the family quality of life (FQOL) among Saudi Arabian families with members who have an intellectual disability. Through the analysis of differences in FQOL perceptions across family roles and the impact of the age of individuals with disabilities, this study sheds light on the nuanced challenges and dynamics within these families.

Table 2 presents descriptive statistics, emphasizing the varied perceptions of family quality of life (FQOL) among different family members relative to individuals with disabilities in all three domains. For example, for the first domain, “family interaction”, the mean FQOL scores reported were 3.498 for fathers, 3.819 for mothers, and 4.098 for other relatives, with standard deviations of 1.131, 1.235, and 0.909, respectively. Post hoc statistics indicate significant differences in the level of satisfaction with FQOL among family members, particularly highlighting that fathers had the lowest satisfaction ratings in comparison with other members in this study sample, however, their ratings were not did not significantly differ from mothers. For example, the family interaction domain revealed a mean difference of −0.600 (SE = 0.250), with a t-value of −2.403, leading to a statistically significant result (*p* = 0.046, *p* < 0.05). This indicates that fathers report significantly lower levels of FQOL compared to other relatives, underscoring the distinct challenges and perceptions fathers may hold within these family dynamics. Conversely, the differences between fathers and mothers (2), and between mothers and other relatives, did not reach statistical significance, with *p*-values exceeding the threshold for significance. This refined analysis highlights the unique position of fathers in experiencing lower satisfaction with FQOL, suggesting a critical area for targeted support and intervention to address their specific needs and improve overall family well-being in the context of intellectual disability. 

Table 3 presents multiple regression results for three distinct domains: Family Interaction, Parenting, and Emotional Well-being. The variables included in the analysis are disability severity, relatives, and age of the person with a disability. Focusing on the statistically significant correlations at *p* < 0.01, the analysis highlights that the age of the individual with a disability significantly affects family interaction (t = 3.195, *p* < 0.01). 

As regards the parenting domain, the relative’s relationship to the member with intellectual disability has a significant association with perceptions in the parenting domain (t = 2.698, *p* = 0.008 *).

As regards the emotional domain, the severity of disability (mild M = 3.74, moderate M = 3.68, and severe M = 3.06) has a significant association with the emotional well-being domain (t = −2.701, *p* = 0.008 *), indicating that higher disability severity is associated with lower emotional well-being among family members.

## 4. Discussion

The aim of this study was to explore the lived experiences of Saudi families navigating the complexities of raising youth with intellectual disabilities. By employing the Beach Center Family Quality of Life Scale, we examined the emotional well-being, family interactions, and parenting within these unique family structures. Our research underscores the intricate dynamics at play, highlighting variations in family quality of life perceptions, particularly among fathers. This study could contribute to filling a critical gap in our understanding of the specific impacts of intellectual disability on Saudi families, and could also serve as a catalyst for developing culturally sensitive interventions aimed at bolstering the well-being of these families. Through this exploration, we aim to contribute valuable insights to the broader dialogue on supporting families with members with disabilities, reinforcing the necessity for targeted and nuanced support strategies.

The results showed that there are no significant differences between fathers’ and mothers’ satisfaction with the level of family quality of life. This is consistent with the findings of Wang et al. [15] when examining mothers’ and fathers’ perceptions of family quality of life. In addition, this study shares similarities with many studies [15,31,32,33] in emphasizing the significant roles both fathers and mothers play in the family dynamics surrounding children with disabilities. 

The finding that there is no significant difference between mothers’ and fathers’ satisfaction with family quality of life is particularly noteworthy. It suggests that both parents share similar experiences and levels of satisfaction, which may reflect the shared responsibilities and emotional bonds in caregiving. It suggests that both parents are likely to experience similar stressors, joys, and coping mechanisms, reinforcing the need for interventions and support programs that consider the entire family unit rather than focusing solely on one parent. This shared satisfaction also implies that any interventions or support systems designed to enhance family quality of life should be inclusive of both parents. Policies and programs should provide resources and support that cater to the needs of both mothers and fathers. By doing so, these interventions can better address the holistic needs of the family, promoting a more balanced and supportive caregiving environment. 

In addition, the result indicating that fathers were less satisfied with the level of family quality of life (FQOL) compared to other family members is a significant finding. This variance might suggest that differences in emotional bonds, involvement, and coping mechanisms across family members impact their views on parenting challenges and successes. Specifically, it highlights how the nuanced experiences of fathers, as discussed earlier in the paper, contribute to the complex dynamics of family interactions and the perceived quality of life, emphasizing the need for nuanced support strategies tailored to each family member’s unique perspective and experience.

In addition, this suggests a unique perspective of fathers in the context of family dynamics when a member has a disability. It might reflect the specific challenges and stressors fathers face, which could be different from those experienced by other relatives, such as brothers and sisters. Understanding these differences is crucial for developing targeted support strategies that cater to the distinct needs of fathers. In Saudi Arabia’s traditional Islamic culture, fathers, often seen as family protectors, might have more leeway to voice dissatisfaction. Our study finds fathers uniquely report lower satisfaction with family quality of life, challenging the notion that men would be more satisfied due to their societal status. This study reveals that fathers are deeply affected by the challenges of raising a child with an intellectual disability, reflecting a broader, more complex picture of family dynamics. It underscores the importance of culturally sensitive support for all family members, highlighting the need to address every individual’s emotional and psychological well-being, including fathers.

This insight also opens up an avenue for further research to explore why fathers feel this way and how their experiences and expectations of FQOL differ from other family members. As there is a justifiable focus on mothers in many studies in the literature (for example, [34,35,36]), such understanding can contribute to more effective family support programs, ensuring that all members, including fathers, receive the appropriate resources and assistance to enhance their overall quality of life. 

This study found that the age of the family member with a disability was associated with a more positive perspective. This result leads us to believe that families with young children with disabilities, particularly in the early stages of dealing with a member with intellectual disability (ID), might require additional support. This also indicates that as the age of the individual with a disability increases perceptions of family interaction improve significantly. Acknowledging the significant correlation between the age of the member with intellectual disability and the time since diagnosis, the positive association between the PWD’s age and improved family perceptions of quality of life can be attributed to the family’s adaptation over time. As families spend more years living with and understanding the disability, they likely develop more effective coping mechanisms, communication strategies, and a deeper acceptance of the condition. This prolonged adaptation period allows families to adjust their expectations, roles, and interactions to better support the member with intellectual disability, leading to a more positive perception of family interaction quality as the member with intellectual disability ages. The strong correlation between the age of the disability and the time of its discovery supports this explanation, indicating that the passage of time plays a crucial role in how families perceive and experience quality of life in relation to caring for a member with intellectual disability. The same results indicate that the longer time since the families discovered that their child had an ID the more positive association they might have. 

One of the key findings from this study is that the severity of disability might impact how families perceive their quality of life. The inverse relationship between disability severity and emotional well-being among families can be attributed to the increased caregiving demands, financial burdens, and social isolation often associated with more severe disabilities. These challenges can significantly affect the emotional and psychological health of family members, leading to stress, anxiety, and a decrease in overall well-being. Given the central role of the family in caregiving within the Saudi Arabian context, these findings underscore the critical need for supportive interventions that are tailored to the severity of the disability.

### 4.1. Limitation

This study might face limitations worth noting. Firstly, its sample specificity in Saudi Arabia may not fully represent the experiences of Arabic families with members who have intellectual disabilities in other countries, suggesting a more nuanced than comprehensive understanding within the Arabic context. Yet, the selection of a focused sample aims to deepen the understanding of Saudi families’ experiences. Additionally, the reliance on self-reported data through the Beach Center Family Quality of Life Scale introduces subjectivity, as participants’ responses might not fully capture the objective realities of family quality of life, despite it being a widely used scale in the field. The choice of variables, including the degree of disability, relatives, and age, was informed by their potential impact on family quality of life, as previously evidenced in the literature. However, acknowledging the limitations of our study, future research should aim to incorporate a wider range of variables to derive more in-depth results and provide a more comprehensive understanding of factors affecting FQOL in families of individuals with intellectual disabilities. 

### 4.2. Implications

The findings of this study have several implications. The lack of significant difference between mothers’ and fathers’ satisfaction with family quality of life highlights the need for inclusive support programs that cater to both parents, reflecting their shared caregiving roles. Additionally, the lower satisfaction reported by fathers compared to other relatives suggests the necessity for targeted interventions addressing fathers’ unique challenges. The positive association between the age of the child with a disability and improved family interaction underscores the importance of early and ongoing intervention services to support families as they adapt over time. Finally, the inverse relationship between the severity of the disability and the family’s emotional well-being indicates that families facing more severe disabilities require more intensive, specialized support. These insights advocate for comprehensive, culturally sensitive strategies to enhance the well-being of Saudi families with youths with intellectual disabilities. 

## 5. Conclusions

In conclusion, this study makes a significant contribution to the field by highlighting the experiences of Saudi families of people with intellectual disabilities. It highlights the crucial roles fathers play alongside mothers, challenges traditional perceptions, and underscores the importance of age and family dynamics in shaping family quality of life. This study’s main findings highlight significant variations in family quality of life perceptions among family members, particularly noting that fathers reported lower levels of satisfaction compared to other relatives. It also underscores the positive association between the age of the youth with an intellectual disability and improved perceptions of family interaction, suggesting that time and adaptation play key roles in enhancing family dynamics. The inverse relationship between disability severity and emotional well-being among families, attributable to increased caregiving demands, financial burdens, and social isolation associated with more severe disabilities, emphasizes the need for culturally sensitive support strategies. These insights emphasize the need for culturally sensitive support strategies that address the unique challenges faced by Saudi families, advocating for inclusive approaches that consider the perspectives of all family members.

## Figures and Tables

**Table 1:** Sample demographics.

Variables		Counts	Total	Proportion
* Relationship to the youth with intellectual disability (ID)	Mother	44	148	0.297
	Father	61	148	0.412
	other	43	148	0.291
Parents’ marital status	Parents are together	121	148	0.82
	Parents are not together	27	148	0.18
Educational background (mother)	Graduate studies	3	148	0.02
	Bachelor	37	148	0.25
	High school	36	148	0.24
	Less than high school	40	148	0.27
	Not educated *	32	148	0.22
Educational background (Father)	Graduate studies	6	148	0.04
	Bachelor	53	148	0.36
	High school	41	148	0.28
	Less than high school	32	148	0.22
	Not educated *	15	148	0.10
** ID severity	Mild	44	148	0.297
	Moderate	81	148	0.547
	Sever	23	148	0.155
The person with a disability age	Mean = 17.1 (Min = 12 years old, Max = 30 years old)			

Note. Proportions tested against value: 0.5, * = participant’s relationship to the youth with ID, ** = the severity of the disability of the member with ID.

**Table 2:** Descriptive statistics by participant’s relativity.

				95% Confidence Interval Mean		
Domains		Valid	Mean	Upper	Lower	Standard Deviation
Family Interaction	Fathers	47	3.498	3.860	3.137	1.131
	Mothers	40	3.819	4.182	3.457	1.235
	Others	39	4.098	4.393	3.804	0.909
Parenting	Fathers	46	3.306	3.682	2.930	1.175
	Mothers	40	3.714	4.069	3.360	1.195
	Others	39	4.069	4.336	3.802	0.824
Emotional well-being	Fathers	46	3.406	3.751	3.062	1.077
	Mothers	40	3.520	3.825	3.215	1.027
	Others	39	3.929	4.216	3.643	0.883

**Table 3:** Multiple regression results for all three domains.

Domains	Variables **	Unstandardized	Standard Error	t	*p* *
Family Interaction	Disability severity	−0.144	0.148	−0.974	0.332
	Relatives	0.223	0.123	1.819	0.071
	Age (PWD)	0.071	0.022	3.195	0.002 *
Parenting	Disability severity	−0.083	0.148	−0.561	0.576
	Relatives	0.333	0.123	2.698	0.008 *
	Age (PWD)	0.046	0.023	2.002	0.048
Emotional	Disability severity	−0.360	0.133	−2.701	0.008 *
	Relatives	0.196	0.111	1.770	0.079
	Age (PWD)	0.053	0.020	2.607	0.010

* *p* < 0.01, PWD = Individual with disability, ** = independent variables.

## Data Availability

The data presented in this study are available on request from the corresponding author. The data are not publicly available due to privacy concerns.
